# A report of the social determinants of health workshop: Muddle cleared up in a polylogue

**DOI:** 10.1002/jgf2.561

**Published:** 2022-06-02

**Authors:** Gemmei Iizuka, Junki Mizumoto, Maho Haseda, Yuya Yokota

**Affiliations:** ^1^ Seibo International Catholic Hospital Family Medicine Residency Tokyo Japan; ^2^ Department of Social Preventive Medical Sciences, Center for Preventive Medical Sciences Chiba University Chiba Japan; ^3^ Department of Medical Education Studies, International Research Center for Medical Education, Graduate School of Medicine The University of Tokyo Tokyo Japan; ^4^ Department of Social Epidemiology, Graduate School of Medicine and School of Public Health Kyoto University Kyoto Japan; ^5^ Family Practice Center of Okayama Okayama Japan

## Abstract

This letter illustrated our online workshop about clinical practice and postgraduate education about social determinants of health (SDH) to family medicine residents and attending physicians in Japan. The participants were encouraged not only by acquiring knowledge and skills but by sharing their experience and talking reflectively. The opportunities for family physicians in Japan to learn about SDH and reflect on their practices should be warranted.

To the Editor,

Health and well‐being are formed by social and economic factors known as the social determinants of health (SDH).^1^ Recognizing and addressing the impacts of SDH on patients should be a vital component of postgraduate medical education.^2^ However, the previous study found that family physicians may be under stress when dealing with patients in social and economic difficulties,^3^ and an educational approach for mitigating the stress is needed.

In February 2022, we held the online workshop at the Family Medicine Winter Seminar for Young Doctors entitled “Patient Care related to Social Determinants of Health: Muddle Cleared up in a Polylogue” in Japan. A total of 22 participants (19 residents and 3 attending physicians) attended this workshop. Its purpose was (i) to alleviate residents' difficulties such as not knowing what to do about SDH in clinical settings or having no one to consult, and (ii) to alleviate attending physicians' difficulties in SDH education. The participants were asked in advance to learn the basic knowledge by reading an SDH educational portal site.^4^ This site contains various educational materials on SDH and a clinical tool kit for addressing patients' social difficulties. We began our workshop with a short presentation about the theory and practice of education and learning on SDH. Second, the participants were asked to share their experiences of difficult encounters with marginalized patients or patients with complex needs and subsequent coordination of care in small groups, followed by peer advice. Third, a lecture on how to address SDH in clinical practice, introducing the framework from “downstream” data to “upstream” advocacy,^1^ was delivered. Last, the participants discussed how to deal with their cases with each other and reflected on their practices.

All the 22 participants completed the post‐questionnaire, and the average scores (standard deviation) on a 5‐point Likert scale (1: strongly dissatisfied to 5: strongly satisfied) were 4.8 (0.4) for clinical relevance, 4.8 (0.4) for clinical usefulness, and 4.8 (0.4) for self‐efficacy, predicting a high level of subsequent practice. The comments from the participants can be summarized as two themes. First, the participants appreciated that they gained knowledge and clinical tools to deal with SDH. Second, the participants had the desire to talk and share their experiences. Many participants reported exposure to complexity and uncertainty in confronting patients with social difficulties but having few opportunities for discussion and reflection. Through small group reflection, they realized that they had been able to tackle their difficult cases, and that they could do more by further understanding and addressing SDH.

Reflection could increase residents' comfort with challenging situations by encouraging them to explore difficulties safely and prepare for future encounters.^5^ This workshop provided residents an opportunity of reflection about SDH in their clinical setting. The attending physicians also learned about knowledge and skills they need to encourage residents' reflection. To help residents address patients' social difficulties without exhaustion and provide comprehensive and contextualized care, we believe that opportunities for continuous learning and reflection regarding SDH need to be provided. Our next goal is to produce regular opportunities for learning and reflection about SDH, and to evaluate participants' progress in addressing SDH in clinical settings.

## CONFLICT OF INTEREST

The authors have stated explicitly that there are no conflicts of interest in connection with this article.
